# Multiple Linear Regressions by Maximizing the Likelihood under Assumption of Generalized Gauss-Laplace Distribution of the Error

**DOI:** 10.1155/2016/8578156

**Published:** 2016-12-07

**Authors:** Lorentz Jäntschi, Donatella Bálint, Sorana D. Bolboacă

**Affiliations:** ^1^Department of Physics and Chemistry, Faculty of Materials and Environmental Engineering, Technical University of Cluj-Napoca, Muncii Boulevard No. 103-105, 400641 Cluj-Napoca, Romania; ^2^Doctoral School of Chemistry, Institute for Doctoral Studies, Babeş-Bolyai University, Kogălniceanu Street No. 1, 400084 Cluj-Napoca, Romania; ^3^Department of Chemistry, Faculty of Science, University of Oradea, Universităţii Street No. 1, 410087 Oradea, Romania; ^4^Department of Medical Informatics and Biostatistics, Faculty of Medicine, Iuliu Haţieganu University of Medicine and Pharmacy, Louis Pasteur Street No. 6, 400349 Cluj-Napoca, Romania; ^5^Doctoral School, University of Agricultural Sciences and Veterinary Medicine Cluj-Napoca, Calea Mănăştur No. 3-5, 400372 Cluj-Napoca, Romania

## Abstract

Multiple linear regression analysis is widely used to link an outcome with predictors for better understanding of the behaviour of the outcome of interest. Usually, under the assumption that the errors follow a normal distribution, the coefficients of the model are estimated by minimizing the sum of squared deviations. A new approach based on maximum likelihood estimation is proposed for finding the coefficients on linear models with two predictors without any constrictive assumptions on the distribution of the errors. The algorithm was developed, implemented, and tested as proof-of-concept using fourteen sets of compounds by investigating the link between activity/property (as outcome) and structural feature information incorporated by molecular descriptors (as predictors). The results on real data demonstrated that in all investigated cases the power of the error is significantly different by the convenient value of two when the Gauss-Laplace distribution was used to relax the constrictive assumption of the normal distribution of the error. Therefore, the Gauss-Laplace distribution of the error could not be rejected while the hypothesis that the power of the error from Gauss-Laplace distribution is normal distributed also failed to be rejected.

## 1. Introduction

The first report on multiple linear regression appears on 1885 [1] and was detailed in 1886 [2]. The classical treatments of the multiple regressions were built on the product-moment method implemented in 1846 [3] and later connected with the optimal correlation [4].

In his first published paper, Fisher introduces the method of likelihood maximization [5], later used in conjunction with Pearson's correlation [6]—a paper which started a contradictory debate between the method of central moments and the method of likelihood estimation [7] replied to in [8] and finally linked with the partial correlation coefficients [9].

A multiple linear regression model involves more than two variables, one (*y*) being assumed dependent and the others (*x*_1_, *x*_2_,…, *x*_*m*_) being assumed to be independent, and is considered here as a continuation of a previous study [10]. The most important assumption is that the data are paired; for example, a natural association between the values of the variables exists. This kind of association is accomplished when for instance a multiple linear regression is constructed involving a measured property/activity (*y*) for a series of compounds for which other compounds measured properties/activities or structure-based descriptors are available (*x*_1_, *x*_2_,…, *x*_*m*_), the natural association being in this case the (chemical) compound responsible for that property/activity/descriptor value.

The least squares method is the standard approach for regression analysis, the method being credited to Legendre [11] (for a debate about the inventor, please see [12]), which also (implicitly) assumes that the error is normally distributed.

Iteratively applying local quadratic approximation to the likelihood (through the Fisher information [13]), the least squares method was used to fit a generalized linear model as a way of unifying classical, logistic, and Poisson (linear) regression in [14] by iteratively reweighing the least squares method in the way to the maximum likelihood estimation of the model parameters.

Generalized Gauss-Laplace distribution is the natural extension [15] from Gauss's [16] and Laplace's [17] symmetric distributions. It is a triparametric distribution (location, scale, and shape) and parameter estimation via maximum likelihood and the method of moments have been reported in [18], concluding that the estimates do not have a closed form and must be obtained numerically.

A more general result regarding the maximum likelihood estimation can be found in [19] but unfortunately provides only conditions in which maximum likelihood estimate exists and is unique without providing the reciprocal (namely, there exist also other conditions than the one given, in which maximum likelihood estimate exists and is unique). Even more, for numerical estimates, it is hardly to discuss unicity.

The problem of estimating the parameters of a multiple linear regression under assumption of generalized Gauss-Laplace distribution of the error is a hard problem which can be solved only numerically and it involves an optimization problem with *m* + 3 constrains, where *m* is the number of unknown (to be determined) coefficients of the multiple linear regression. In this paper a mathematical and a numerical treatment of the problem is proposed.

In order to provide a proof of the facts for the proposed method of relaxing the distribution of the error when linear regression is used to link between chemical information and biological measurements, ten previously reported datasets were considered, all with significant role in human medicine or ecology.

## 2. Mathematical Treatment

One may define the generalized Gauss-Laplace (GL) distribution as

(1) GLx;μ,σ,q=q2σΓ1/23/qΓ3/21/qexp⁡−x−μ/σqΓ1/q/Γ3/qq/2⁡,

where Γ(·) is the Gamma function and *μ* (location), *σ* (scale), and *q* (shape) are the parameters of the distribution.

This definition will be used here for the Gauss-Laplace distribution to relax the normal distributed constraint for the distribution of the error (*ε*).

### 2.1. Statement of the Problem

Multiple linear regressions under assumption of GL distribution (see (1), for the error *ε*; (*a*_*j*_)_1≤*j*≤*m*_, *σ* and *q* are to be determined from sampled data) are stated in the following equation:

(2) $$\begin{array}{c} \text{MLRGL}\left(\;\varepsilon ;\sigma ,q\;\right)=\text{GL}\left(\;\varepsilon ;{\left(\;a_{j}\;\right)}_{1\leq j\leq m},\sigma ,q\;\right), \end{array}$$

where *ε* = *y* − ∑_1≤*j*≤*m*_ *a*_*j*_ *x*_*j*_ (and $\hat{\varepsilon }\approx 0$ and $\overset{-}{\varepsilon }=0$).

The case with intercept ($y\approx \hat{y}=a_{0}+\sum_{1\leq j\leq m}^{}a_{j}x_{j}$) is reduced to the case without intercept by increasing with one the number of the independent variables (*a*_*m*+1_ ← *a*_0_; *x*_*m*+1_ ← 1; and *m* ← *m* + 1, when $y\approx \hat{y}=\sum_{1\leq j\leq m+1}^{}a_{j}x_{j}$) and therefore will not be mentioned further. The substitution given as (2) transforms the distribution from univariate to a multivariate one and can be mathematically characterized by a series of properties, such as is given in [29] (results applicable resizing *x* from 0 and *x*_0_ ← *y*).

Let us take a sample of *n* paired measurements (e.g., (*y*_*i*_, (*x*_*j*,*i*_)_1≤*j*≤*m*_)_1≤*i*≤*n*_, where *n* is the number of paired measurements and *m* is the number of independent measures). The likelihood for the sample is

(3) $$\begin{array}{c} \text{LMLRGL}\left(\;\cdot \;\right)=\overset{n}{\underset{i=1}{\sum }}\ln\left(\;\text{MLRGL}\left(\;\cdot \;\right)\;\right). \end{array}$$

Doing the substitution given in (2) and expressing in full its parameters, the expression of the likelihood function from (3) becomes

(4) LMLRGLaj1≤j≤m,σ,q=n·ln⁡q2σΓ1/23/qΓ3/21/q−σqΓ1/qΓ3/qq/2∑i=1nyi−∑1≤j≤majxj,iq.

The likelihood is at maximum when all its partial derivatives are zero:

(5) 0∂∂a1LMLRGLaj1≤j≤m,σ,q=⋯=∂∂qLMLRGLaj1≤j≤m,σ,q.

### 2.2. Simplification of the Problem

The problem of finding the maximum of the likelihood is a typical problem of finding the extreme points, but not easy to be solved because it depends on a large number of variables. The easiest way is to eliminate one variable, namely, *σ*. The derivative of LMLRGL by *σ* provides the value of *σ*:

(6) n=q·Sσq·Γ1/q/Γ3/qq/2,Sq,aj1≤j≤m=∑i=1nyi−∑1≤j≤majxj,iq.

Please note that *S* = *S*(*q*, (*a*_*j*_)_1≤*j*≤*m*_) does not depend on *σ*. Therefore, let LMLRGLS(*S*, *n*, *q*) be the function having this constraint. After some calculations, the expression of LMLRGLS(*S*, *n*, *q*) is

(7) LMLRGLS,n,qnqq·ln⁡n1/qq1−1/q2S1/q·Γ1/q−1

(8) SSq,aj1≤j≤m=∑i=1nyi−∑j=1majxj,iq=∑i=1nTi,aj1≤j≤mq/2,

where *T*(*i*, (*a*_*j*_)_1≤*j*≤*m*_) = (*y*_*i*_ − ∑_1≤*j*≤*m*_ *a*_*j*_ *x*_*j*,*i*_)^2^.

On the other hand, only *S* depends on (*a*_*j*_)_1≤*j*≤*m*_, and therefore when the derivative of *S* relative to *a*_1_,…, *a*_*m*_ is zero, then also the derivative of the maximum likelihood estimation function (either any of LMLRGL and LMLRGLS) is zero. Doing the partial derivatives of *S*, with the above given substitution (function *T*), the following equation is the result:

(9) ∑i=1nyixu,iWi,q=∑j=1maj·∑i=1nxu,ixj,iWi,q;for  1≤u≤m,

where *W*(*i*, *q*) = (*y*_*i*_ − ∑_1≤*j*≤*m*_ *a*_*j*_ *x*_*j*,*i*_)^*q*−2^.

At this point only the expression of the likelihood function (see (7) and (8)) must be included in the new statement of the problem (see (9)) in order to keep in full the derivatives constraints of the initial problem (see (4) and (5)). There is no obvious further reduction of the problem. However, revising the results obtained till this point, the cancelling of the (likelihood function) derivative relative to *σ* was included at the beginning of the simplification (see (6)) while the cancelling of the (likelihood function) derivatives relative to the regression coefficients *a*_1_,…, *a*_*m*_ is equivalent to the previous given equation for the regression coefficients (see (9)). On the other hand, (7)–(9) facilitate an iterative solution of the problem.

## 3. Fixed-Point Theory for Iterating to the Optimal Solution

A convenient notation was used in (9) to suggest the further treatment of the problem. Actually, Fisher and Mackenzie proposed for the first time to use such numerical treatment in statistics [30]. This is based on the assumption that, near to the optimal solution, an iterative evaluation of the coefficients (here *q* and (*a*_*j*_)_1≤*j*≤*m*_) conducted using their previous values (hidden in (9) inside of the function *W*(*i*, *q*)) leads to the optimum. The optimum is obtained when no significant change from one step to another is on their values, and, at that time, the *W*(*i*, *q*) function acts as the argument of a contraction mapping [31].

There are some inconveniences for a smooth application of the fixed-point theory. One of them is that the obtaining of the maximum of the LMLRGLS function (see (7), being obtained for known values of *S* and *n* (where (∂/∂_*q*_)LMLRGLS(*S*, *n*, *q*) vanishes)) is not a very simple problem; it is expected from its explicit expression to have more than one local maximum. Fortunately, some clues exist, such as the domain of *q* (ranging from 0) and the expectance from the power of the error (here let us say that it is expected to have *q* from 0.1 to 10 and is very unlikely but possible to have *q* from 0.01 to 100, but outside of this range also precision of computations often fails). But the biggest inconvenience is that (9) is not an equation, but a system of equations, and here we may only provide different strategies of iteration, hoping that at least one of them provides the contraction mapping. Namely, we may

(i) start from some initial values of the regression coefficients ((*a*_*j*,0_)_1≤*j*≤*m*_) and for the power of the error *q*_0_;
(ii) use initial values to obtain the likelihood function LMLRGLS (from (7)) as a function depending only on *q*; it requires only the evaluation of *S* (see (8));
(iii) find the maximum (let this be *ϑ*) of LMLRGLS function from (7) (where its derivative is 0 and the point is a global extreme point);
(iv) prepare starting of a loop on *k*, by setting it to 0 (*k* ← 0);
(v) it is possible, especially at the beginning of the iteration (when *k* = 0), that *ϑ* and *q*_*k*_ be largely different one to each other; a major change in *q* will accelerate the convergence but will also increase the likelihood of divergence; therefore, use the new (*ϑ*) and old (*q*_*k*_) value to indicate the gradient of the change in *q*, such as *q*_*k*+1_ ← *δ* · *q*_*k*_ + (1 − *δ*) · *ϑ*, with small 0 < *δ* < 1 to be determined;
(vi) do a loop (*k* ← *k* + 1) using the new value of *q* (namely, *q*_*k*+1_) to calculate the new values of the coefficients ((*a*_*j*,*k*+1_)_1≤*j*≤*m*_ with (9)) and using the new values of the coefficients calculate the new value of *q* (turn back to find the maximum from (7));
(vii) repeat until ((*a*_*j*,*k*+1_)_1≤*j*≤*m*_) and (*q*_*k*+1_) have no changes during iteration.

At arriving in the stationary point, all criteria for the maximization of the likelihood are accomplished; namely, the equations corresponding to all derivatives cancellation are assured. The great advantage of this proposed method is that it reduces the problem of finding the maximum of a function with *m* + 3 variables to the finding of the maximum of a function with one variable (*q*), in a repeated process, of course.

The disadvantage is that the evolution is through a contracting functional of which contraction cannot be assured all the time. This is the reason why there are different strategies of finding such kind of contracting functional (see example  6.1 in [32] for construction of a contracting functional from resampling).

Some calculations are the same regardless the strategy used and are given in the next as Algorithms 1 [Fig alg2]–3.

One strategy is to use the equations from cancellation of regression coefficients derivatives (see (9)) to iterate the values of the coefficients, while another one is to treat (9) as a system of equations and to solve it as a whole (Algorithm 4).

Another strategy that is required to be specified is that if (7)–(9) is used to simply iterate for new values or if (9) should be used in a loop to converge to new estimates for the coefficients associated with the new *q* (Algorithm 5). The expected assumption is that the errors are normally distributed (*q* = 2) and the optimal solution of (5) is near to this.

The contingency of 2 × 2 strategies given above was tested on sampled data (see Section 4), and the pair (Algorithms 4 and 5) turned out to be the only one providing a contraction functional. Thus, for convenience, the working algorithm is given in full (see Algorithm 6) and was used to obtain the results given in the next section.

In order to assure the numerical stability of the calculations, Algorithm 6 was used with fixed and reasonable value *ε* = 10^−5^, and in order to assure a smooth convergence, the value of the new error's power estimate (*q*_*j*_) was replaced by an exponential smoothing (a technique commonly applied to time series [33]), *q*_*j*_ ← 0.1 · *q*_*j*_ + 0.9 · *q*_*j*−1_.

Therefore, in all scenarios, the initial (starting) values of the estimates to be determined will be the one given by the classical multiple linear regression models as presented in the following:

(10) q0⟵2;ak,01≤k≤m⟵MLRy,x1,…,xk,MLRy,x1,…,xk=xTx−1xTy,

where MLR(*y*, *x*_1_,…, *x*_*k*_) uses the classical strategy of ordinary least squares ((**x** ^*T*^ **x**)^−1^ **x** ^*T*^ **y**) to find the parameters.

## 4. Case Study

Ten datasets of chemical compounds with different sample size (Table 1) along with their measured outcome activity were considered to illustrate Algorithms 1 [Fig alg2] [Fig alg3] [Fig alg4] [Fig alg5]–6.

For all datasets, the experimental values of the dependent variable (*y*) and for the selected previously reported independent variables (under the assumption of the normal distribution of the error) on multiple linear regressions with two (*m* = 2) independent variables are given in Table 2.

Different descriptors (independent variables) were used to explain the activity/property of interest on models presented in Table 2. The names of these descriptors are

(i) TIE: state topological parameter [20];
(ii) TIC1: total information content index (neighborhood symmetry of 1) [20];
(iii) IHDMkMg and IHDDFMg: MDF descriptors [21];
(iv) SAG: molecular surface area grid; *f*(0)_*n*_: Fukui index [22]
(v) TPSA(NO): topological polar surface area expressed by nitrogen and oxygen contributions; Aeigm: Dragon descriptor [23];
(vi) RDF035m: radial distribution function on a spherical volume of a 3.5 Å radius weighted by atomic mass; small-RSI-mol: the smallest value of atomic steric influence in a molecule [24];
(vii) ^0^ *χ* ^*v*^: Kier's molecular connectivity index; *μ*: dipole moment [25];
(viii) nR10: number of 10-membered rings; N-070: number of Ar-NH-Al fragments [26];
(ix) N_Cl_: the number of the chlorine atoms on the two phenyl rings; *V*_*s*_^+^: the surface maxima values of the electrostatic potential; and *σ* ^2^_tot_: total variance of the electrostatic potential at a point *r*_*i*_ [27];
(x) FNSA1: fractional partial positive surface area 1 PPS_*A1*_/TMA_*S*_; where PPS_*A*_ = Partial Positive Surface Area and TMS_*A*_ = Total Molecular Surface Area.

All sets subjected to analysis converged maximizing the likelihood and Table 3 provides the differences between values obtained by classical MLR approach and values obtained by the proposed approach (MLR-MLE-GL).

The results presented in Table 3 reveal different estimates for the coefficients in the assumption of the more general generalized Gauss-Laplace distribution of the error. In 6 out of 10 cases, the power of the error proved to be higher compared with convenient value of 2, the highest values being observed for set 10 (*q* = 2.937). Opposite, the power of the error proved to be almost half of the expected value for set 5 (*q* = 1.058). The values of coefficients obtained by applying the MLE and the proposed approach were close to each other in two cases (set 4 and set 8). With one exception, represented by set 2, the sum of the absolute differences of *a*_0_, *a*_1_, and *a*_2_ was less than 1. The values obtained for the population standard deviation by the two investigated methods proved to be closest to each other, with highest difference of 0.49310 observed on set 10.

**Figure 1:**
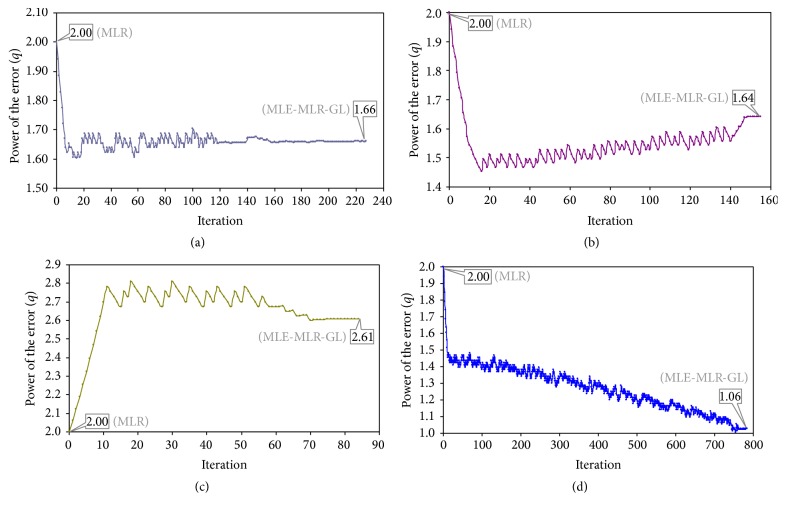
Evolution of the power of the errors (*q*) by optimization iteration: (a) set 1 (converged at 226); (b) set 6 (converged at 154); (c) set 8 (converged at 83); and (d) set 5 (converged at 784).

The power of the error follows different patterns according to the model, decrease-fluctuation-plateau (set 1, Figure 1(a)), decrease-increase in steps-plateau (set 6, Figure 1(b)), increase-fluctuation-decrease in steps-plateau (set 8, Figure 1(c)), and decrease-fluctuation (set 5, Figure 1(d)).

A question (hypothesis) can be raised about the power of the error:* if its distribution can be assumed normal*. This hypothesis (the distribution of the power of the error can be assumed to be normal) can be tested on the results even if the sample is small (10 cases) to provide an answer. However, the tendency to have a mean of two in convergence is clear ($\overset{-}{q}=2.06$ from the 10 cases) and the hypothesis of its normality cannot be rejected (Anderson-Darling statistic measures that only 14.72% (*p*_to-reject_ = 0.8528 > 0.05) of the random samples are in better agreement with the normal distribution while Kolmogorov-Smirnov statistic measures only 28.7% (*p*_to-reject_ = 0.713 > 0.05)).

## 5. Conclusions

The proposed algorithm (Algorithm 6 in this paper) was found to provide an appropriate contraction mapping to be used for maximum likelihood estimation of the multiple linear regression parameters in the generalized Gauss-Laplace distribution assumption of the measurement's errors. The analysis conducted on 10 samples demonstrated that, in general, it is not appropriate to assume that the measurement error is normally distributed, and when it is possible a deeper treatment of the distribution of the error need to be conducted. From a sample of 10 cases, the analysis of the distribution of the error showed that the normal distribution of the power of the error could not be rejected, being very likely to have a mean equal to two.

## Figures and Tables

**Algorithm 1:**
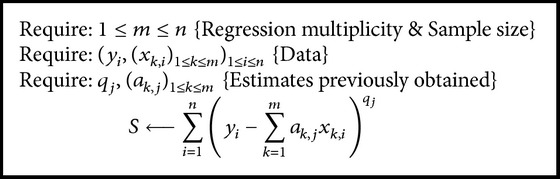
Calculate “*S*” at some step “*j*” from (8).

**Algorithm 2 alg2:**
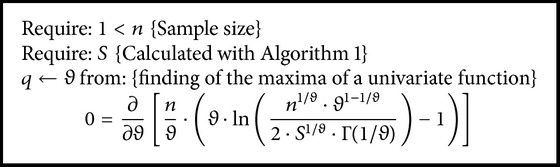
Calculate “*q*” at some step “*j*” from (7).

**Algorithm 3 alg3:**
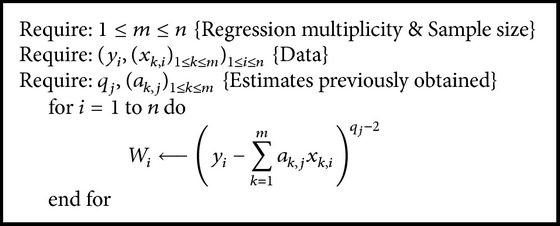
Calculate “(*W*_*i*_)_1≤*i*≤*n*_” at some step “*j*” from (9).

**Algorithm 4 alg4:**
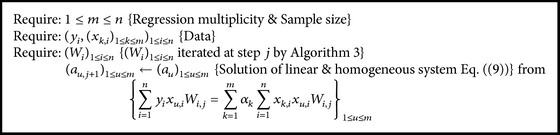
Block solves providing “(*a*_*u*,*j*+1_)_1≤*u*≤*m*_” at some step “*j*” with (9).

**Algorithm 5 alg5:**
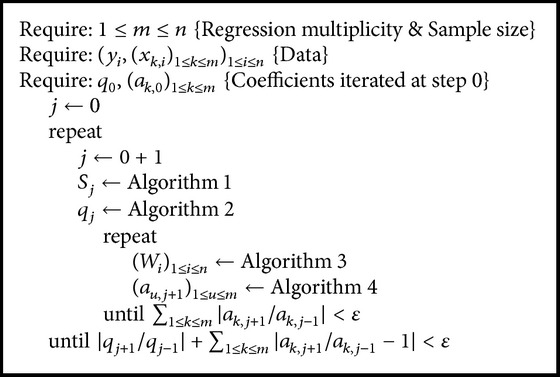
Double loop with (9) for (7) and (8).

**Algorithm 6:**
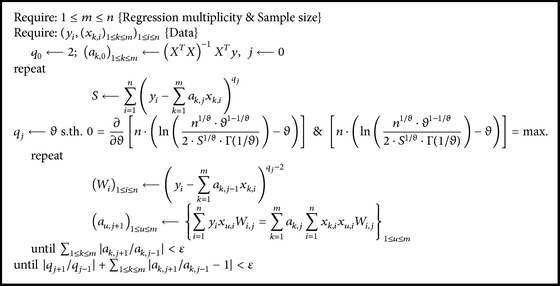
Contraction functional for MLR-MLE-GL.

**Table 1:** Datasets characteristics.

Set	Sample size (*n*)	Class	Property/activity	Reference
1	132	Estrogens	Estrogen binding affinity—log⁡RBA	[20]
2	37	Carboquinone derivatives	Minimum effective dose (MED)—log⁡(1/MED)	[21]
3	33	Organic pollutants	Oxidative degradation—log⁡(*k*′)	[22]
4	97	Benzotriazoles	Fish toxicity—pEC50	[23]
5	136	Thiophene and imidazopyridine derivatives	Inhibition of polo-like kinase 1—pIC_50_	[24]
6	14	Substituted phenylaminoethanones	Average antimicrobial activity—pMICam	[25]
7	110	Acetylcholinesterase inhibitors	Inhibition activity—pIC_50_	[26]
8	107	Polychlorinated biphenyl ethers	298 K supercooled liquid vapor pressures—log⁡(*p*_*L*_)	[27]
9	107	Polychlorinated biphenyl ethers	Aqueous solubility—log⁡(*S*_*w*_, *L*)	[27]
10	47	Para-substituted aromatic sulphonamides	Carbonic anhydrase II inhibitors—log⁡(*K*_*i*_)	[28]

**Table 2:** Reported bivariate models.

Set	Model under assumption of normal errors	Determination coefficient (*r* ^2^)
1	−4.284 − 0.0263 · TIE + 0.0368 · TIC1	0.3976
2	7.780 − 579 · IHDMkMg + 0.049 · IHDDFMg	0.7700
3	−2.703 + 0.00515 · SAG + 9.703 · *f*(0)_*n*_	0.6859
4	4.110 − 0.0172 · TPSA(NO) + 0.0097 · Aeigm	0.7161
5	2.5651 + 0.1899 · RDF035m + 2.9825 · Small-RSI-mol	0.5101
6	0.780 + 0.0339 · ^0^ *χ* ^*v*^ + 0.004 · *μ*	0.8357
7	5.446 + 0.716 · nR10 + 1.113 · N-070	0.6838
8	1.476 − 0.588 · N_Cl_ − 5.029 · 10^−2^ · *V*_*s*_^+^	0.9880
9	−4.080 − 0.880 · N_Cl_ + 5.996 · *σ* ^2^_tot_	0.9619
10	4.055 − 0.154 · ^0^ *χ* ^*v*^ − 1.284 · FNSA1	0.7058

**Table 3:** Differences between values of coefficients obtained by classical linear regression approach compared to the proposed approach.

Set	diff(*q*)	diff(*a*_0_)	diff(*a*_1_)	diff(*a*_2_)	diff(LMLRGL)	diff(*σ*)
1	0.3400	−0.00073	−0.00315	0.24400	−0.30000	−0.00200
2	−0.4150	−0.00034	−16.30000	0.17400	−0.10100	−0.00020
3	−0.3830	−0.28700	0.00009	−0.04000	−0.06000	−0.00030
4	−0.1680	0.00006	0.00007	−0.01400	−0.05000	0.00000
5	0.9420	0.34500	−0.00880	−0.62400	−6.10000	−0.00850
6	0.5000	0.00027	0.00078	−0.02140	−0.09000	−0.00006
7	0.5210	−0.10300	0.03490	−0.01800	−1.10000	0.00030
8	−0.5690	0.00090	−0.00330	−0.01100	−0.42000	−0.00010
9	−0.4370	−0.27700	−0.00020	0.04000	−0.30000	−0.00020
10	−0.9370	0.01400	−0.00700	0.06000	−0.70600	0.49310

diff: difference between value obtained by classical approach and value obtained by the proposed approach.

*a*_0_, *a*_1_, and *a*_2_: coefficients of the independent variables; *q*: power of the error (Algorithm 6 for the proposed approach).

*σ*: population standard deviation; LMLRGL: likelihood for multiple linear regressions under assumption of GL distribution.
